# Fiber-utilizing capacity varies in *Prevotella*- versus *Bacteroides*-dominated gut microbiota

**DOI:** 10.1038/s41598-017-02995-4

**Published:** 2017-06-01

**Authors:** Tingting Chen, Wenmin Long, Chenhong Zhang, Shuang Liu, Liping Zhao, Bruce R. Hamaker

**Affiliations:** 1 State Key Laboratory of Microbial Metabolism and Ministry of Education Key Laboratory of Systems Biomedicine, School of Life Sciences and Biotechnology, Shanghai Jiao Tong University, Shanghai, 200240 China; 2 Whistler Center for Carbohydrate Research, Department of Food Science, Purdue University, 745 Agriculture Mall Drive, West Lafayette, IN 47907 USA

## Abstract

The gut microbiota of individuals are dominated by different fiber-utilizing bacteria, which ferment dietary fiber into short chain fatty acids (SCFAs) known to be important for human health. Here, we show that the dominance of *Prevotella* versus *Bacteroides* in fecal innocula, identified into two different enterotypes, differentially impacts *in vitro* fermentation profiles of SCFAs from fibers with different chemical structures. In a microbiome of the *Prevotella* enterotype, fructooligosaccharides, and sorghum and corn arabinoxylans significantly promoted one single *Prevotella* OTU with equally high production of total SCFAs with propionate as the major product. Conversely, in the *Bacteroides*-dominated microbiota, the three fibers enriched different OTUs leading to different levels and ratios of SCFAs. This is the first report showing how individual differences in two enterotypes cause distinctly different responses to dietary fiber. Microbiota dominated by different fiber-utilizing bacteria may impact host health by way of producing different amounts and profiles of SCFAs from the same carbohydrate substrates.

## Introduction

Human gut microbiota varies among individuals and the concept of “enterotypes” has been used to stratify people’s microbiota compositions. Different enterotypes are defined by their dominant bacteria, and microbiota dominated by *Prevotella* and *Bacteroides* genera have been observed^1, 2^. The health relevance of these enterotypes remains to be elucidated. *Prevotella* and *Bacteroides*-dominated microbiota are associated with long-term dietary patterns, with the former associated with complex carbohydrate consumption and the latter associated with plenty of protein and animal fats^2, 3^. *Prevotella* has been associated with non-industrialized populations whose diets contain more dietary fiber, such as African (Burkina Faso) children^4^, Hadza hunter-gatherers^5^, and the people of the Amazonas of Venezuela and rural Malawi^6^. *Bacteroides* is more prevalent in Western populations who consume higher animal-based diets.

Both *Prevotella* and *Bacteroides* are well known as dietary fiber fermenters. Their high abundance in each enterotype provides an opportunity to understand how the behavior of the dominant species affects the whole community, and how this influences the production of short chain fatty acids (SCFAs) which are important for host health. Among the SCFAs, butyrate is a preferred energy source for the colonocytes and further promotes barrier function and reduces inflammation^7, 8^. Propionate is metabolized in the liver and decreases hepatic lipogenesis and reduces serum cholesterol, and is potent in triggering the enteroendocrine L-cells to signal a satiety response^9^. Acetate is primarily used as an energy source, but also recently was shown to have a circulating peripheral effect at the arcuate nucleus of the hypothalamus to reduce appetite^10^. These SCFAs are biologically important in amount and proportion.

Since SCFAs are biologically active compounds produced by fiber-utilizing bacteria, it becomes an interesting question whether different enterotypes produce different amounts and profiles of SCFAs from the same fiber substrates. If true, this could be a way how different enterotypes are involved in host health. In this study, SCFAs were used as the functional endpoint. Two microbiota, one high in *Prevotella* and the other high in *Bacteroides*, were used to study how different enterotypes utilize dietary fibers of different chemical structures.

## Results and Discussion

**Figure 1:**
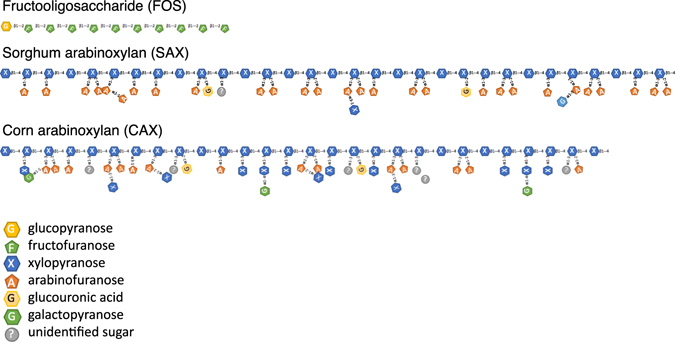
Illustration of the fiber structures of fructooligosaccharides (FOS), sorghum arabinoxylan (SAX), and corn arabinoxylan (CAX).

**Figure 2:**
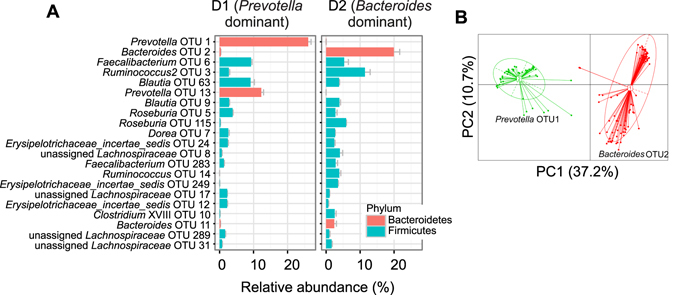
Fecal sample microbiota compositions for D1 and D2. (**A**) Relative abundance of OTUs with abundance larger than 1% in at least one sample. (**B**) Clustering of microbiota from D1 and D2 together with 54 healthy subjects shows their alignment with *Prevotella* and *Bacteroides* enterotypes, respectively.

Previously, we found that structurally different dietary fibers within the arabinoxylan fiber class were utilized differently using *in vitro* human fecal fermentation^11^. Arabinoxylans from sorghum bran (SAX) fermented rapidly compared to corn arabinoxyan (CAX) which fermented slowly. The structural differences between the two arabinoxylans arise from branched structures where SAX has relatively uniform arabinose substitution on the xylan backbone and CAX has a more complex array of sugars and linkage patterns (Fig. 1, Table S1). Their molecular sizes were similar (Table S2). In the current study, we found in microbiota samples from various individuals that the two arabinoxylans were either utilized differently, as observed before, or were fermented at the same rate (Fig. S1). This was the basis for the selection of two donors (D1 and D2) which had divergent microbiomes. Microbiota of D1 was dominated by *Prevotella* (OTU1 and OTU13) at 38.7%, and D2 by *Bacteroides* (OTU2 and OTU 11) at 25.0% (Fig. 2A). The microbiota compositions of these two donors were analyzed together with fecal microbiota sequences from a cohort of 54 healthy Chinese subjects and two disparate clusters were observed (Fig. 2B), which aligned with the *Prevotella* (D1) and *Bacteroides* (D2) enterotypes as put forth by Arumugam^1, 2^. These two enterotypes were then used to study how the two microbiota communities utilized the different fibers.

**Figure 3:**
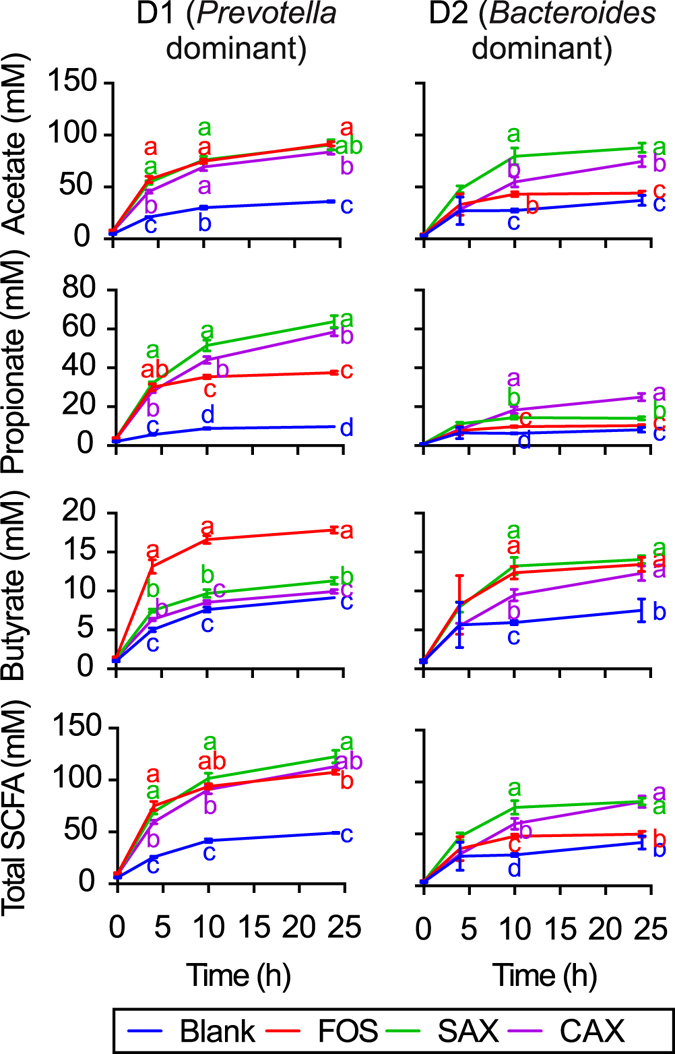
Short chain fatty acid (SCFA) *in vitro* fermentation products in D1 and D2 when fed fructooligosaccharides (FOS), sorghum arabinoxylan (SAX), and corn arabinoxylan (CAX). Different letters indicate significant differences in SCFAs among fiber treatments at the same time point (α = 0.05).

The D1 *Prevotella* enterotype microbiota fermented all three fibers with similar high total SCFA production (Fig. 3). Compared to FOS, CAX and SAX produced similarly high levels of propionate and low levels of butyrate. The three fibers produced the same levels of acetate. Conversely, the D2 *Bacteroides* enterotype microbiota showed differential fermentation of CAX and SAX with different SCFA outcomes. The more complicated CAX structure was slower fermenting and initially produced less total SCFAs, though by 24 h reached the same high level as SAX. The rapid initial rise in SCFAs of SAX was contributed by higher acetate and butyrate production. Surprisingly, FOS which is generally recognized as a fast fermenting fiber^12^, generated significantly less total SCFAs than SAX and CAX by 24 h, with little production of acetate.

The *Prevotella*-dominated microbiota produced 2–3 times more propionate than the *Bacteroides*-dominated microbiota. Overall, the two microbiota fermented the carbohydrate structures differently to produce different amounts and ratios of the SCFAs.

**Figure 4:**
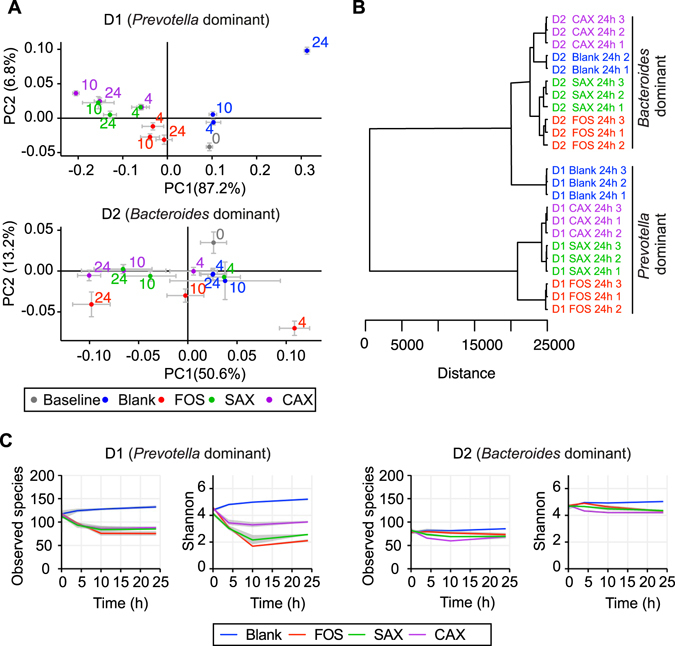
Microbiota composition shift for D1 and D2 over the 24 h fermentation. (**A**) PCoA plot of jackknifed weighted UniFrac distance within the bacteria community shift under different treatments and times. Numbers by the symbols indicate fermentation time (h). Mean values ± SD are plotted. (**B**) Dissimilarity of microbiota after 24 h fermentation with each fiber. Samples were clustered using the Ward agglomerative algorithm on Euclidean distances. Distances were calculated using arbitrary units. (**C**) Observed species and Shannon diversity change over time.

**Figure 5:**
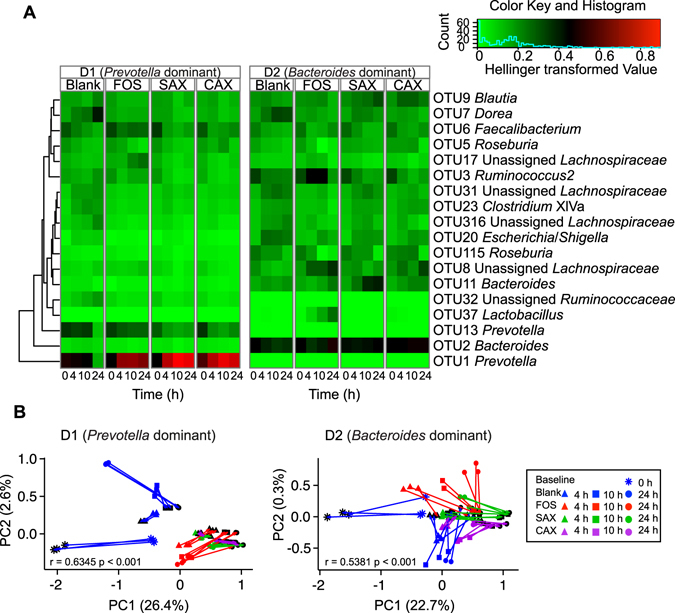
Microbiota shifts during fermentation and correlation with SCFA production. (**A**) Heatmap of the shift in key OTUs during fermentation. Key OTUs picked out by RDA ordination biplot based on the Hellinger-transformed Euclidean distance among samples (Legendre *et al*.^24^) and the eigenvalue of each OTU. The color of the heatmap from green to red represents the abundance of each OTU after the Hellinger transformation (square root of each OTU abundance divided by mean OTU abundance of each sample). Mean value of three replicates are plotted. (**B**) The Jaccard distance of SCFA compositions (end of lines with black symbols) to the microbiota compositions (end of lines with colored symbols) as superimposed by Procrustes. Two symbols connected by a line indicate two data sets from the same sample. P-values were calculated by PROTEST, p < 0.001 indicates significant concordance of the two PCoAs.

We then examined the changes in the microbiomes during fermentation of the different fibers in the two enterotypes. The structure of the microbiota shifted significantly during the 24 h fermentation for the three fiber treatments based on principal coordinate analysis with UniFrac distance (Fig. 4A). Cluster analysis showed clear separation of each donor’s microbiota and the effects of each fiber treatment (Fig. 4B). The exception was for the D1 *Prevotella*-dominated microbiota on basal culture medium containing peptides (Blank), where at 24 h there was a large shift to *Dorea* and *Blautia* which are known to be promoted by protein^11^ (Fig. S2). α-Diversity (Fig. 4C) decreased significantly during fermentation in D1, but not in D2, even though D1 had more observed species at 0 h. This seems to have occurred because *Prevotella* in D1 was virtually the only bacteria to increase on the fiber treatments. In D1, redundancy analysis (Fig. S3) identified *Prevotella* OTU1 as the most positive responder to the fiber treatment, and this OTU alone was further increased during fermentation by FOS, SAX, and CAX (Fig. 5A). In contrast, in D2, nine OTUs were identified as responders by RDA analysis that had comparable contribution to the microbiota shift (Fig. S3). FOS increased *Ruminococcus2* OTU3 and *Lactobacillus* OTU37, SAX increased *Bacteroides* OTU2, and CAX increased *Bacteroides* OTU11 (Fig. 5A). In the D2 *Bacteroides* enterotype, there was clearly more diversity and response of bacteria to the fiber substrates than in the D1 *Prevotella* enterotype (Fig. 5A). Procrustes analysis (r = 0.63 for D1 and r = 0.54 for D2, p < 0.001) on Jaccard distance matrices of the microbiota and SCFA compositions showed that the structural shifting of the gut microbiota was highly correlated with individual SCFA production (Fig. 5B).

Higher total SCFA production of the *Prevotella* compared to *Bacteroides* enterotype indicates higher fiber utilizing capacity, and is a reasonable outcome of plant-rich diets that are associated with the *Prevotella* enterotype. The dominant *Prevotella* OTU1 in D1 effectively outcompeted the other major bacteria for use of the three fibers (FOS, CAX, SAX), and diminished their growth. *Prevotella spp*. are commonly found in herbivores such as in cattle and sheep, as well as the human gut, and are known to harbor the enzymes for degrading an array of polysaccharides, including xylans^13^. Kovatcheva-Datchary *et al*.^14^ recently reported individuals with *Prevotella*-dominated gut microbiota responded to barley kernel bread supplementation with improved glucose tolerance. This implies an enterotype-dependent beneficial effect of dietary fibers.

These markedly different SCFA outcomes from fiber fermentation in the *Prevotella* and *Bacteroides* enterotypes relate to different preference and capacity of species to ferment different fiber structures. Therefore, in the interest of designing dietary fiber treatments for specific types and amounts of SCFAs, the enterotype of the individual should be considered. By testing fibers in an *in vitro* fermentation system, we charted the relationship between microbiota composition and capacity to utilize certain fibers. It seems conceivable that with the accumulation of more information on how fibers affect different human gut microbiota, guidance for future personalized dietary fiber recommendations or therapies can be developed based on enterotypes.

## Materials and Methods

### Study design

Fecal samples were obtained from participants who met the following criteria: ages 18 to 50, no history of gastrointestinal disease, no antibiotic usage within the previous 6 months. This study was approved by Institutional Review Board of Purdue University (protocol #1509016496) and the Ethics Committee of Shanghai Jiao Tong University. All experiments were performed under guidelines and regulations of the IRB protocol. Informed consent was obtained from all donors. Six participants recruited at Shanghai Jiao Tong University (healthy Chinese) provided fresh stool samples on site on the day of the fermentation. Participants were requested to fill out a 24 h dietary recall form and a food frequency questionnaire. A screening procedure was applied to differentiate microbiota by how they fermented two arabinoxylans of different structural complexity. Two donors were chosen as D1 to represent no difference in fermentation profiles between SAX and CAX, and D2 as a large difference in profiles coupled with a high initial rate of fermentation for SAX. The two participants were asked to provide stool samples for the study. All participants were requested to keep a normal dietary pattern and life style, no excessive alcohol drink during the study.

### Fiber preparation

Fructooligosaccharide (FOS) was obtained from Beneo HP Orafti (Tienen, Belgium); corn and sorghum arabinoxylans (CAX, SAX) were extracted from brans; corn bran was a gift from Bunge Milling (St. Louis, MO, USA), and sorghum grain (from the Purdue Agronomy Center for Research and Education) was decorticated using an abrasive dehuller and the bran part collected. Arabinoxylans were extracted from corn and sorghum brans as described previously^11^. Alkaline hydrogen peroxide was employed to improve the arabinoxylan yield, reduce the lignin content, and reduce the dark color. Briefly, brans were defatted using hexane at room temperature. Then starch and protein were removed by α-amylase and protease at optimal temperature and pH, respectively. After removing the fat, starch and protein, the bran was stirred in 1 M NaOH at 60 °C for 4 h, and 15 mL of 30% hydrogen peroxide was slowly added to the mixture. After cooling down, the mixture was centrifuged at 10,000 g for 10 min. Supernatants were collected, and residues were washed with 1 M NaOH and centrifuged again. The combined supernatants were neutralized with 6 M HCl, the arabinoxylans were precipitated by adding 4 volumes of 95% ethanol, and then freeze-dried and ground into a powder form.

### *In vitro* fecal fermentation and metabolite analyses

Fermentations were conducted as described in Long *et al*.^15^. Fresh stool samples were obtained from participants and immediately transferred into an anaerobic chamber. Three volumes of basic culture medium were added into the samples and vortexed until dispersed. Slurries were filtered through four layers of cheesecloth to remove visible particles. Fecal inocula (1%) and 1% (w/v) of a solution each of FOS, CAX, and SAX were mixed with the culture medium [peptone water (2 g/L), yeast extract (2 g/L), NaCl (0.1 g/L), K_2_HPO_4_ (40 mg/L), KH_2_PO_4_ (40 mg/L), MgSO_4_·7H_2_O (10 mg/L), CaCl_2_·6H_2_O (10 mg/L), NaHCO_3_ (2 g/L), L-cysteine (0.05%), bile salts (0.5 g/L), vitamin K (10 μl/L), Tween 80 (2 ml/L), and hemin (5 mg/L), adjusted to pH 7.4]^15^. The mixtures were incubated at 37 °C and, at 0, 4, 10, and 24 h, gas production was measured using a syringe, and culture samples were collected and centrifuged. Supernatants were filtered through sterilized syringe filters with a polyethersulfone membrane of 0.22 μm pore size for SCFA measurements. SCFA analysis was performed by gas chromatography as described by Kaur *et al*.^12^. Significant differences among the SCFAs generated by fermentation were analyzed using two-way ANOVA followed by Tukey’s Multiple Comparison test.

Fecal samples are representative of luminal bacteria in the descending colon, though are limited in reflecting the mucosal microbiota. Although some mucosal bacteria are observed in stools^16^, data obtained from *in vitro* fecal fermentations may not represent the mucosal bacterial changes that would occur under the same fiber treatment *in vivo*.

### DNA extraction and sequencing

The pellets were used for DNA extraction. DNA was extracted as previously described^17, 18^. Purification, amplification, and pyrosequencing were conducted following the procedure of Zhang *et al*.^18^. Briefly, extracted DNA was purified using a QIAamp DNA mini kit (QIAGEN, Germany), and the V1–V3 region of the 16 S rRNA gene was amplified by PCR. The primers were 5′-CGTATCGCCTCCCTCGCGCCATCAGACGAGTGCGTAGAGTTTGATYMTGGCTCAG-3′ and 5′-CTATGCGCCTTGCCAGCCCGCTCAGNNNNNNNNNNATTACCGCGGCTGCTGG-3′ with a sample-unique 10-mer oligonucleotide barcode. Equal amount of the PCR products for each sample were mixed and subjected to pyrosequencing using the GS FLX platform (Roche, Branford, CT, USA).

### Bioinformatics analysis

Raw sequences, obtained from the platform, were filtered to obtain high quality reads using the criteria described in Zhang *et al*.^18^. Sequences were then analyzed using the UPARSE pipeline^19^. Briefly, the sequences were trimmed to 300 bases, dereplicated, and then clustered into OTUs (Operational Taxonomic Units, closely related bacterial sequences with 97% similarity approximately associated with species). Chimeras were removed using Genomes OnLine Database (GOLD) from UPARSE as the reference. An OTU table was created through mapping reads back to OTUs. The taxonomy of each OTU was assigned by blasting representative OTU sequences against the Ribosomal Database Project (RDP) Naive Bayesian rRNA Classifier (rrnDBv4.2.2)^20^. The confidence threshold was set at 80%. After obtaining the OTU table, the QIIME platform was used^21^. Singleton OTUs and samples with abnormally low number of reads were removed. The microbiota from D1 and D2 were subject to cluster analysis, together with 54 healthy subjects. The Calinski-Harabasz index (*CH*) was used to evaluate the cluster validity based on the average between and within the cluster sum of squares. From this analysis, the subjects were grouped into two clusters. The microbiota percentage composition was summarized at the genus level. α-Diversity, using observed species and Shannon metrics, was calculated using QIIME scripts. Jackknifed weighted UniFrac metrics^22^ were calculated to evaluate β-diversity, and principal coordinate analyses were performed. Samples were then evened to 1,000,000 sequences per sample to eliminate differences caused by different amounts of sequences per sample. Dissimilarities among samples were clustered by hierarchical cluster analysis using the Ward agglomerative algorithm on Euclidean distances. For construction of the heatmap, key OTUs were chosen through redundancy analysis using Vegan package in R^23^. Redundancy analysis (RDA) is a form of constrained ordination that examines how much of the variation in one set of variables explains the variation in another set of variables. It is the multivariate analog of simple linear regression. The mean values of the Hellinger-transformed OTU abundances for each treatment were plotted. The distance between samples is called the Hellinger distance, which is a measure recommended for clustering or ordination of species abundance data and offers a better compromise between linearity and resolution than the chi-square metric and the chi-square distance^24^. OTUs were grouped by hierarchical clustering. The similarity of the SCFA compositions and microbiota compositions were analyzed by Procrustes analysis (Vegan package in R). In statistics, Procrustes analysis is a form of statistical shape analysis used to analyze the distribution of a set of shapes. The Jaccard distance of SCFAs and microbiota compositions were calculated. Jaccard distance is a statistical method used for comparing the similarity and diversity of sample sets. The two ordinations were superimposed by Procrustes analysis and the significance of correlation was calculated by Protest.

## Electronic supplementary material

Supplementary info

## References

[1] Arumugam M (2011). Enterotypes of the human gut microbiome. Nature.

[2] Wu GD (2011). Linking long-term dietary patterns with gut microbial enterotypes. Science.

[3] Lim MY (2014). Stability of gut enterotypes in Korean monozygotic twins and their association with biomarkers and diet. Sci. Rep..

[4] De Filippo C (2010). Impact of diet in shaping gut microbiota revealed by a comparative study in children from Europe and rural Africa. Proc. Natl. Acad. Sci. USA.

[5] Schnorr SL (2014). Gut microbiome of the Hadza hunter-gatherers. Nat Commun.

[6] Yatsunenko T (2012). Human gut microbiome viewed across age and geography. Nature.

[7] Hamer HM (2008). Review article: the role of butyrate on colonic function. Aliment. Pharmacol. Ther..

[8] Leonel AJ, Alvarez-Leite JI (2012). Butyrate: implications for intestinal function. Curr. Opin. Clin. Nutr. Metab. Care.

[9] Chambers ES (2015). Effects of targeted delivery of propionate to the human colon on appetite regulation, body weight maintenance and adiposity in overweight adults. Gut.

[10] Frost G (2014). The short-chain fatty acid acetate reduces appetite via a central homeostatic mechanism. Nat Commun.

[11] Rumpagaporn P (2015). Structural features of soluble cereal arabinoxylan fibers associated with a slow rate of *in vitro* fermentation by human fecal microbiota. Carbohydr Polym.

[12] Kaur A, Rose DJ, Rumpagaporn P, Patterson JA, Hamaker BR (2011). *In vitro* batch fecal fermentation comparison of gas and short-chain fatty acid production using “slowly fermentable” dietary fibers. J. Food Sci..

[13] Flint HJ, Bayer EA, Rincon MT, Lamed R, White BA (2008). Polysaccharide utilization by gut bacteria: potential for new insights from genomic analysis. Nat. Rev. Microbiol..

[14] Kovatcheva-Datchary P (2015). Dietary Fiber-Induced Improvement in Glucose Metabolism Is Associated with Increased Abundance of Prevotella. Cell Metab..

[15] Long W (2015). Differential responses of gut microbiota to the same prebiotic formula in oligotrophic and eutrophic batch fermentation systems. Sci. Rep..

[16] Eckburg PB (2005). Diversity of the Human Intestinal Microbial Flora. Science.

[17] Godon JJ, Zumstein E, Dabert P, Habouzit F, Moletta R (1997). Molecular microbial diversity of an anaerobic digestor as determined by small-subunit rDNA sequence analysis. Appl. Environ. Microbiol..

[18] Zhang C (2012). Structural resilience of the gut microbiota in adult mice under high-fat dietary perturbations. ISME J.

[19] Edgar RC (2013). UPARSE: highly accurate OTU sequences from microbial amplicon reads. Nat. Methods.

[20] Wang Q, Garrity GM, Tiedje JM, Cole JR (2007). Naive Bayesian classifier for rapid assignment of rRNA sequences into the new bacterial taxonomy. Appl. Environ. Microbiol..

[21] Caporaso JG (2010). QIIME allows analysis of high-throughput community sequencing data. Nat. Methods.

[22] McMurdie PJ, Holmes S (2014). Waste not, want not: why rarefying microbiome data is inadmissible. PLoS Comput. Biol..

[23] Oksanen, J. *et al*. *vegan*: *Community Ecology Package*. *R package version 2*.*2*-*1*., http://cran.r-project.org/package=vegan (2015).

[24] Legendre P, Gallagher E (2001). Ecologically meaningful transformations for ordination of species data. Oecologia.

