# Supplementary material for: Fiber-utilizing capacity varies in Prevotella- versus Bacteroides-dominated gut microbiota
Source: Sci Rep. 2017 Jun 1;7:2594. doi: 10.1038/s41598-017-02995-4 (PMC5453967; doi:10.1038/s41598-017-02995-4)
Supplement: Supplementary file 1 — Supplementary info [file 41598_2017_2995_MOESM1_ESM.pdf]

# Fiber-utilizing capacity varies in *Prevotella*- versus *Bacteroides*-dominated gut microbiota

**Authors:** Tingting Chen[2], Wenmin Long[1], Chenhong Zhang[1], Shuang Liu[1], Liping Zhao[1 *], Bruce R. Hamaker[1, 2 *]

**Supplementary figures**

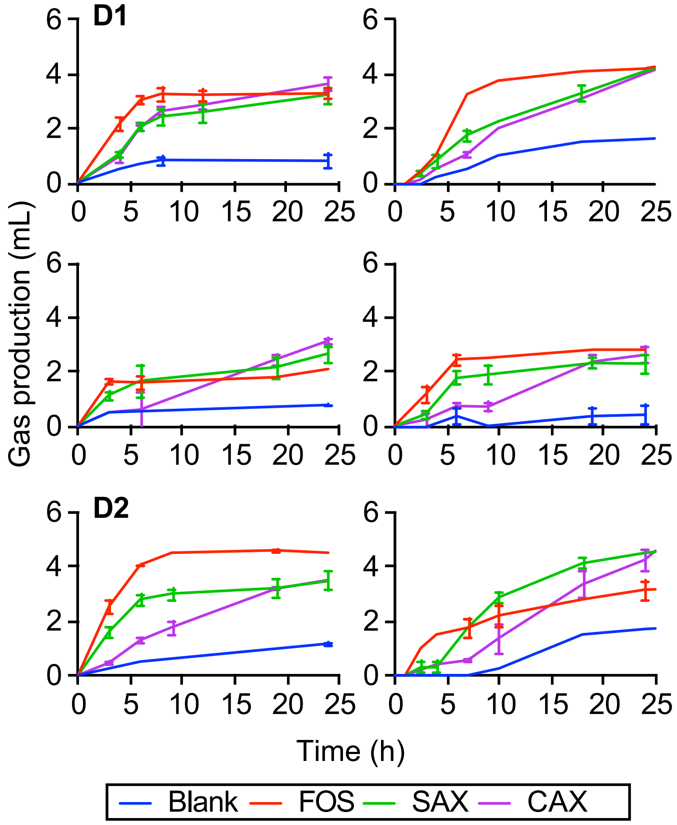


Supplementary Fig. S1. *In vitro* gas production of stools of 6 individuals fermented with fructooligosaccharides (FOS), sorghum arabinoxylan (SAX), and corn arabinoxylan (CAX). Plots arranged (by top left to bottom right) by relative differences of area under curves of SAX and CAX. Donors chosen as D1 to represent no difference in fermentation profiles between SAX and CAX and D2 as large difference coupled with high initial rate of fermentation for SAX.

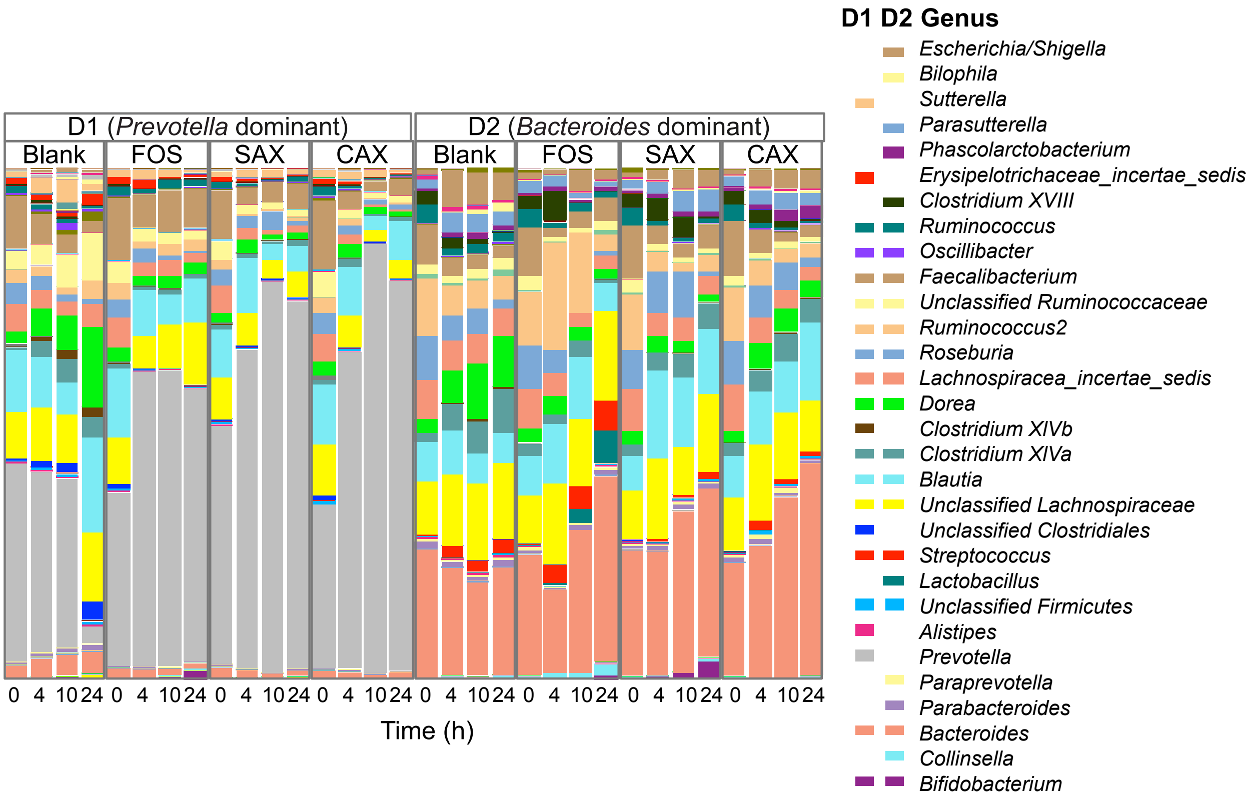


Supplementary Fig. S2. Relative abundance of genus level microbial distribution within each fiber fermentation.

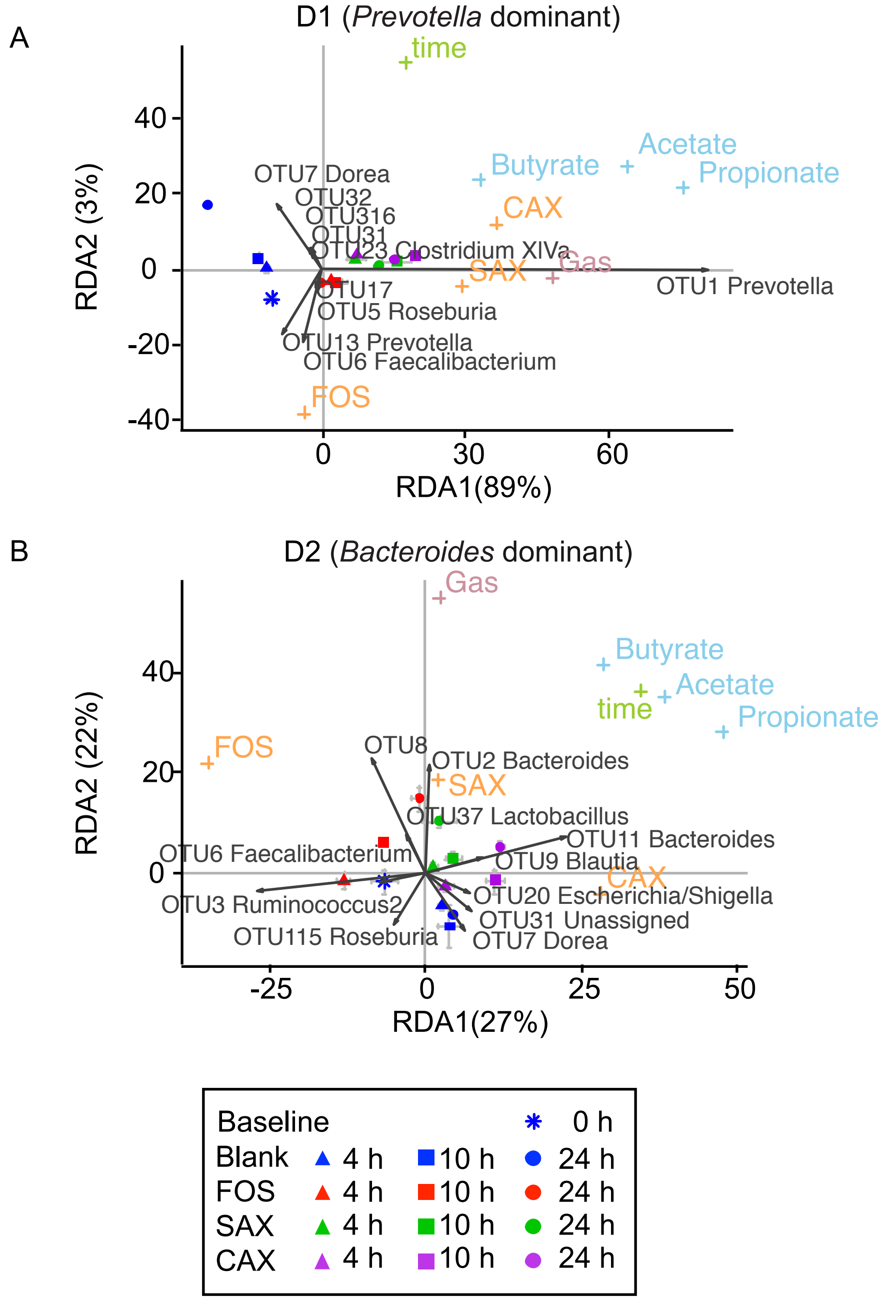


Supplementary Fig. S3. Redundancy analysis (RDA) calculated from complete OTU data and environmental factors based on Euclidean distance. OTUs that significantly contributed to this ordination plot, are shown by arrows. Environmental variables are shown by cross sign. P-values of the Monte Carlo Permutation Procedure (MCPP) are both smaller than 0.001.

**Supplementary Tables**

Supplementary Table S1. The monosaccharide and linkage type composition of sorghum and corn arabinoxylan. Data represented as percentage (%) ± S. D.

| Monosacharides | CAX | SAX | Linkage | CAX | SAX |
|---|---|---|---|---|---|
| **Rhamonose** | 0.22 ± 0.05 | nd | nd | | |
| **Arabinose** | 48.12 ± 0.41 | 32.90 ± 0.65 | T-Ara-f | 15.32 ± 0.98 | 40.10 ± 1.85 |
| | | | T-Ara-p | nd | 2.17 ± 0.61 |
| | | | 2-Ara-f | 7.25 ± 0.21 | 2.71 ± 0.15 |
| | | | 3-Ara-f | 5.71 ± 0.14 | 2.53 ± 0.07 |
| | | | 4-Ara-p | 3.05 ± 0.13 | 2.23 ± 0.84 |
| | | | 5-Ara-f | 10.21 ± 0.15 | 9.96 ± 0.58 |
| | | | 2,3,4-Ara-p | 9.88 ± 0.42 | 9.09 ± 0.57 |
| **Xylose** | 41.02 ± 0.56 | 54.57 ± 1.07 | T-Xyl | 12.67 ± 0.61 | nd |
| | | | 4-Xyl | nd | 1.71 ± 0.18 |
| | | | 3,4-Xyl | 28.73 ± 1.06 | 26.10 ± 0.45 |
| **Galactose** | 3.36 ± 0.17 | 9.59 ± 0.37 | T-Gal | 5.51 ± 0.48 | 1.05 ± 0.18 |
| | | | 2-Gal | 0.73 ± 0.05 | nd |
| **Glucose** | 7.28 ± 0.46 | 2.94 ± 0.16 | 4-Glc | 0.94 ± 0.02 | 2.34 ± 0.08 |

Supplementary Table S2. The retention time (RT), molecular weight (MW) and degree of polymerization(DP) of sorghum and corn arabinoxylan.

| | RT | logMW | MW | DP |
|---|---|---|---|---|
| **FOS** | 73.61 | 2.67 | 471.54 | 2.62 |
| **SAX** | 50.25 | 5.13 | 136071.60 | 755.95 |
| **CAX** | 49.98 | 5.16 | 145404.47 | 807.80 |
